# Toward Understanding Molecular Bases for Biological Diversification of Human Coronaviruses: Present Status and Future Perspectives

**DOI:** 10.3389/fmicb.2020.02016

**Published:** 2020-08-25

**Authors:** Takaaki Koma, Shun Adachi, Naoya Doi, Akio Adachi, Masako Nomaguchi

**Affiliations:** ^1^Department of Microbiology, Tokushima University Graduate School of Medical Science, Tokushima, Japan; ^2^Department of Microbiology, Kansai Medical University, Osaka, Japan

**Keywords:** COVID-19, SARS-CoV-2, SARS-CoV, MERS-CoV, HCoV, biological diversification, recombination, adaptive evolution

## Abstract

Human coronaviruses (HCoVs) are of zoonotic origins, and seven distinct HCoVs are currently known to infect humans. While the four seasonal HCoVs appear to be mildly pathogenic and circulate among human populations, the other three designated SARS-CoV, MERS-CoV, and SARS-CoV-2 can cause severe diseases in some cases. The newly identified SARS-CoV-2, a causative virus of COVID-19 that can be deadly, is now spreading worldwide much more efficiently than the other two pathogenic viruses. Despite evident differences in these properties, all HCoVs commonly have an exceptionally large genomic RNA with a rather peculiar gene organization and have the potential to readily alter their biological properties. CoVs are characterized by their biological diversifications, high recombination, and efficient adaptive evolution. We are particularly concerned about the high replication and transmission nature of SARS-CoV-2, which may lead to the emergence of more transmissible and/or pathogenic viruses than ever before. Furthermore, novel variant viruses may appear at any time from the CoV pools actively circulating or persistently being maintained in the animal reservoirs, and from the CoVs in infected human individuals. In this review, we describe knowns of the CoVs and then mention their unknowns to clarify the major issues to be addressed. Genome organizations and sequences of numerous CoVs have been determined, and the viruses are presently classified into separate phylogenetic groups. Functional roles in the viral replication cycle *in vitro* of non-structural and structural proteins are also quite well understood or suggested. In contrast, those in the *in vitro* and *in vivo* replication for various accessory proteins encoded by the variable 3′ one-third portion of the CoV genome mostly remain to be determined. Importantly, the genomic sequences/structures closely linked to the high CoV recombination are poorly investigated and elucidated. Also, determinants for adaptation and pathogenicity have not been systematically investigated. We summarize here these research situations. Among conceivable projects, we are especially interested in the underlying molecular mechanism by which the observed CoV diversification is generated. Finally, as virologists, we discuss how we handle the present difficulties and propose possible research directions in the medium or long term.

## Introduction

**FIGURE 1:**
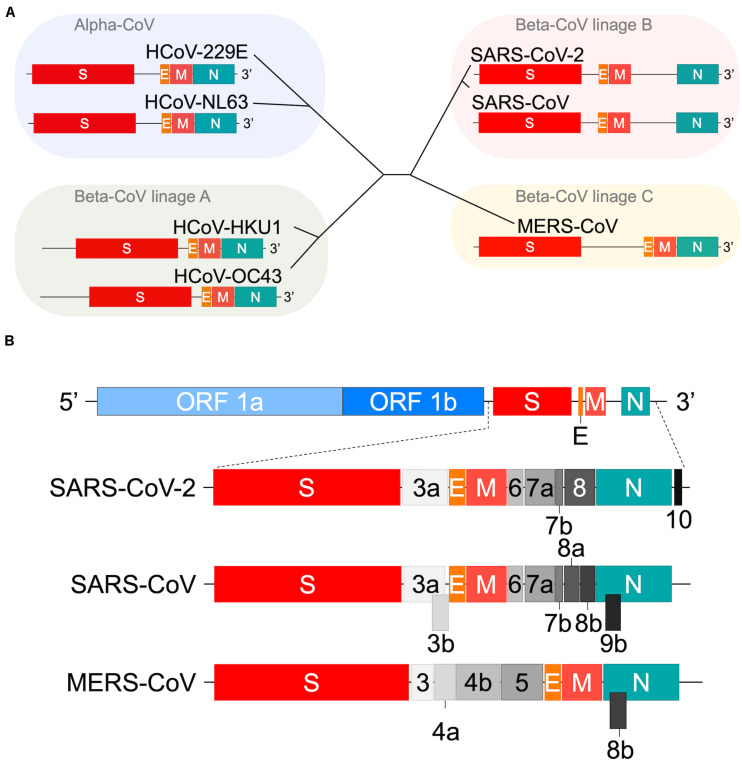
Genome organization of human coronaviruses. **(A)** Schematic representation of the variable 3′ genomic region. The genome organization of viruses in the four viral groups (Su et al., 2016; Forni et al., 2017) including SARS-CoV-2 (Tse et al., 2020; Wang N. et al., 2020) are shown. Those for various accessory proteins are omitted. The HE *orf* sequences of HCoV-HKU1 and HCoV-OC43 (Table 3 footnote) are also omitted. The beta-CoV lineage D group that contains some bat viruses only is not presented in this panel. **(B)** Comparison of SARS-CoV-2, SARS-CoV, and MERS-CoV genomes. All *orf* sequences in the full-length viral genome are shown (Totura and Baric, 2012; Su et al., 2016; Forni et al., 2017; Cui et al., 2019; Biswas et al., 2020; Hou et al., 2020; Thao et al., 2020; Wang N. et al., 2020; Wu A. et al., 2020; Xie et al., 2020). It has been reported that SARS-CoV-2 contains *orf 9b* (Cagliani et al., 2020), suggesting a more similar genome structure between SARS-CoV and SARS-CoV-2 than shown in panel B.

People around the world now have been seeing a global devastating outbreak of COVID-19, caused by a new human coronavirus (HCoV) designated severe acute respiratory syndrome CoV 2 (SARS-CoV-2) (Lu et al., 2020; Wu A. et al., 2020; Zhu et al., 2020). Various CoVs were isolated from mammals and birds, and were long considered to be weakly pathogenic until the identification of SARS-CoV (Drosten et al., 2003; Fouchier et al., 2003; Ksiazek et al., 2003; Zhong et al., 2003) followed by the Middle East respiratory syndrome virus MERS-CoV (Zaki et al., 2012) as a causative virus for serious human infectious disease. Before the three outbreaks, a number of human coronaviruses were discovered and found to be responsible for a seasonally prevalent viral disease with mild symptoms such as the common cold and/or diarrhea (Forni et al., 2017; de Wilde et al., 2018; Cui et al., 2019; Tse et al., 2020; Wang N. et al., 2020; Ye et al., 2020). These include HCoV-NL63 (van der Hoek et al., 2004), HCoV-229E (Hamre and Procknow, 1966), HCoV-OC43 (McIntosh et al., 1967), and HCoV-HKU1 (Woo et al., 2005) in Figure 1. The principal scientific question for virologists and the investigators of other research fields is what makes each CoV or group of CoVs behave so distinctively from the others. Although a large number of excellent articles on the clinical outcomes of COVID-19 and relevant host immune responses have been published very recently (Biswas et al., 2020; Bost et al., 2020; Broggi et al., 2020; Brouwer et al., 2020; Cao et al., 2020; Davies et al., 2020; Giamarellos-Bourboulis et al., 2020; Gordon et al., 2020; Grifoni et al., 2020; Hansen et al., 2020; Ju et al., 2020; Kadkhoda, 2020; Kim D. et al., 2020; Long et al., 2020; McKechnie and Blish, 2020; Oberfeld et al., 2020; Ong et al., 2020; Polycarpou et al., 2020; Robbiani et al., 2020; Shi R. et al., 2020; Subbarao and Mahanty, 2020; Tang D. et al., 2020; Tay et al., 2020; Vabret et al., 2020; Wilk et al., 2020; Xu et al., 2020; Ye et al., 2020; Zhang et al., 2020; Zhou P. et al., 2020; Zhou Z. et al., 2020; Ziegler et al., 2020; Zohar and Alter, 2020), fundamental studies aimed at the above issue have been poorly carried out. Needless to mention, biological and molecular bases for the observed CoV divergence should be elucidated urgently for basic science and clinical applications in the future. As for the origin and evolution of the seven HCoVs (Figure 1) described above, researchers have sufficiently clarified this particular subject by their extensive efforts through field and *in silico* analyses (Su et al., 2016; Forni et al., 2017; Cui et al., 2019; Adachi et al., 2020; Biswas et al., 2020; Tang D. et al., 2020; Ye et al., 2020). However, mechanistic bases for the adaptive mutations to generate distinct virus groups/lineages/clades are insufficiently elucidated as yet. In summary, we have focused on the baseline studies on the HCoV diversification in this review article by picking up on relevant biological and molecular biological issues from previously published reports. The selected subjects should be experimentally and conclusively analyzed by molecular genetic methods of the day to obtain definitive answers.

The most prominent feature of CoVs is their exceptionally large genome RNA (∼30 kb) (Fehr and Perlman, 2015; Forni et al., 2017; Cui et al., 2019; Han et al., 2019; Hou et al., 2020; Thao et al., 2020; Wu A. et al., 2020; Xie et al., 2020). Furthermore, it is single-stranded, non-segmented, and polycistronic. While the conserved 5′ two-thirds of the genome encodes a series of non-structural proteins for the replicase-transcriptase complex, the variable 3′ one-third encodes a variety of structural and accessory proteins (Figure 1). Thus, the regulation for well-timed CoV gene expressions should be quite complicated. Another outstanding characteristic of CoVs is their ability for extremely high genomic recombination (Lai, 1992; Nagy and Simon, 1997; Rowe et al., 1997; Lauring et al., 2013; Su et al., 2016; Forni et al., 2017; Cui et al., 2019; Adachi et al., 2020). Among numerous animal virus species, CoVs are known to be first-class for their recombination frequency (Lai, 1992). Probably consistent with this observation, considerably many *cis*-acting sequences/structures critical for RNA replication and transcription have been identified throughout the coronavirus genome (Fehr and Perlman, 2015). The gene recombination is considered to have dual evolutionary consequences (Simon-Loriere and Holmes, 2011). While it can increase the rate of adaptive evolution by creating advantageous genetic variations, it also can stabilize genomic RNA by generating a functional genome through removing deleterious mutations/deletions. The observed high genomic recombination rates thus confer the plasticity to the CoV genome. Finally, the CoV genome encodes diverse accessory proteins at the variable 3′ one-third portion (Figure 1). These accessory proteins differ in the number and sequence even among the CoVs of the same viral lineage (Forni et al., 2017). They are believed to play a role in suppressing host innate immunity (Cruz et al., 2011; Totura and Baric, 2012; Forni et al., 2017; de Wilde et al., 2018; Cui et al., 2019; Park and Iwasaki, 2020), thus promoting viral adaptation to some specific host species and individuals. Obviously, a precisely organized description of knowns and unknowns about the general picture of HCoV would certainly generate new and significant insights into the corona-virology and shed light on the CoV research today.

The transmission of CoVs between host species (species tropism) and individuals is a major issue to be addressed. The tissue and cell tropism of the viruses within individuals is critically important as well. In general, viral tropism is determined at the surface of target cells by direct binding of the virus and cellular receptor molecule(s) and/or at the post-entry intracellularly replication step(s) (Nomaguchi et al., 2012a, b). As for pathogenic HCoVs, the primary cellular receptors have been identified as angiotensin-converting enzyme 2 (ACE2) for SARS-CoV (Li et al., 2003), dipeptidyl peptidase 4 (DPP4) for MERS-CoV (Raj et al., 2013), and ACE2 for SARS-CoV-2 (Letko et al., 2020a; Lu et al., 2020; Walls et al., 2020; Wu A. et al., 2020; Zhou P. et al., 2020; Zhu et al., 2020). It has been well-established that ACE2 and DPP4 work for the coronaviral receptors and determinants of the coronavirus tropism (Table 1; Fehr and Perlman, 2015; Forni et al., 2017; de Wilde et al., 2018; Biswas et al., 2020; Letko et al., 2020a; Oberfeld et al., 2020; Tang D. et al., 2020; Tse et al., 2020; Wang N. et al., 2020; Zhou P. et al., 2020). It is unclear as yet on the biological and mechanistic bases by which HCoV-NL63 and SARS-CoV/SARS-CoV-2 of two distinct phylogenetic groups use the same receptor ACE2. Many CoVs utilize peptidases as the cellular receptor, despite that their enzymatic domains are not required for the viral entry process (Fehr and Perlman, 2015). It has been reported that the receptor-specific clustering for viral receptor-binding proteins from the family *Coronaviridae* is absent (Ng et al., 2020), and that there exist many HCoV receptors other than ACE2 (Fehr and Perlman, 2015; de Wilde et al., 2018; Ye et al., 2020). These reports suggest a complicated evolutionary pathway for HCoVs, which may include the switch to the same receptor on multiple occasions (Li F. et al., 2005; Wu et al., 2009; Song et al., 2018). Further study is necessary to elucidate this biologically important issue. Notably, some co-factors such as cellular proteases and sialic acids are required for efficient CoV entry into cell cytosols for subsequent viral replication (Fehr and Perlman, 2015; Forni et al., 2017; de Wilde et al., 2018; Oberfeld et al., 2020; Tang D. et al., 2020). Whether there is another/other receptor(s) for CoVs remains elusive (Letko et al., 2020a). Also, whether some unknown cell factor(s) restricts the CoV intracellular replication needs to be determined. These cellular factors may influence viral tropism, replication, transmission, pathogenicity, and thus viral ecology.

**TABLE 1:** Origin and receptor-usage of major human coronaviruses.

Viruses	Genera and lineages	Hosts*	Entry receptors
HCoV-NL63	Alpha-CoV	Bats	ACE2
HCoV-229E	Alpha-CoV	Bats	ANPEP/CD13
HCoV-OC43	Beta-CoV lineage A	Rodents, Bovines	Unknown
HCoV-HKU1	Beta-CoV lineage A	Rodents	Unknown
SARS-CoV	Beta-CoV lineage B	Bats, Palm civets	ACE2
MERS-CoV	Beta-CoV lineage C	Bats, Dromedary camels	DPP4/CD26
SARS-CoV-2	Beta-CoV lineage B	Bats	ACE2

Viruses (upper six) are listed according to the timeline of their emergences previously reported (Forni et al., 2017). For reference, see the relevant review articles (Fehr and Perlman, 2015; Su et al., 2016; Forni et al., 2017; de Wilde et al., 2018; Cui et al., 2019; Biswas et al., 2020; Tang D. et al., 2020; Tse et al., 2020; Wang N. et al., 2020; Ye et al., 2020). HCoV-OC43 and HCoV-HKU1 use sialic acids as co-receptors. Host cell protease furin cleaves SARS-CoV-2 S protein at the virus entry step, suggesting a significant difference in the viral entry mechanism and infectivity compared with those of SARS-CoV (Andersen et al., 2020; Hoffmann et al., 2020; Walls et al., 2020). ACE2, angiotensin-converting enzyme 2; ANPEP, alanyl aminopeptidase; DPP4, dipeptidyl peptidase. *Natural and intermediate animal hosts with a consensus in the research field are shown. It has been reported that camelids and pangolins may be intermediate hosts for HCoV-229E and SARS-CoV-2, respectively (Ye et al., 2020).

Based on the above described considerations, in this review article, we describe and discuss: (i) the integrative virology of HCoVs, (ii) reverse genetics systems for human and animal CoVs, and (iii) conclusion: future studies in a demonstrative and perspective manner. In this challenging time, we, as experimental virologists, need to initiate basic HCoV studies to counteract SARS-CoV-2. While focusing on studies on human and simian retroviruses for a long time, we also have significant research experience in many other viruses. Coronaviruses and retroviruses are virologically distinct, but it is quite clear that the principal purpose, main concept, and major research strategy for current virology are commonly shared among basic researchers. We have summarized important scientific issues from the viewpoint of our own. Here, we aim to concentrate on studies in the medium or long term. First, we outline basic factual matters such as grouping viruses based on their ecology/evolution/pathogenicity, genome organization, replication cycle, and functional aspects of individual viral proteins. We then summarize the applications of the reverse genetics system, a powerful tool regularly used in current virology, to CoVs with an extremely large RNA genome to demonstratively analyze all kinds of viral properties. Finally, as a whole, we present basic research directions against coronaviruses severely pathogenic for humans, which would also lead to the establishment of effective anti-viral strategies against possible re-emerging and emerging viruses of various viral species.

## Integrative Virology of HCoVs

### Classification, Genome Organization, and Basic Properties

Coronavirus is a positive-sense RNA virus [RNA (+) virus] and a member of the family *Coronaviridae*. All coronaviruses have a highly conserved total genome organization and commonly have a specific open reading frame (ORF) structure (Figure 1). Based on extensive sequence comparisons, coronaviruses are divided into four genera, i.e., alpha-CoV, beta-CoV, gamma-CoV, and delta-CoV (Su et al., 2016; Cui et al., 2019; Wang N. et al., 2020; Ye et al., 2020). HCoVs belong to the alpha-CoV genus, or to the beta-CoV genus constituting a major large phylogenetic group. Beta-CoVs are further classified into lineages A, B, C, and D. As clearly observed in Figure 1A, each virus lineage is readily distinguished by its ORF structure. Close examination of the ORF structure of the viral genome has revealed that each virus has its own organization at the 3′ variable genomic region, whereas no variations are found for the 5′ conserved genomic region (Figure 1B). Also, the ORF structure at the 3′ genomic region appears to vary from strain to strain within a viral group (Forni et al., 2017; Cui et al., 2019). Thus, HCoVs have a unique set of genes of their own.

Currently, seven different HCoVs are known as representatives of each distinctive virus group that infects humans as described above in “Introduction” section. Table 1 lists these HCoVs with some virological information. While certain seasonal HCoVs (HCoV-NL63 and HCoV-229E) belong to alpha-CoV and are of bat origin, others (HCoV-OC43 and HCoV-HKU1) belong to beta-CoV and are of rodent origin. In general, these four viruses appear to be well-adapted to humans and broadly circulate among human populations in some countries in specific seasons (Su et al., 2016). As a cellular receptor, while HCoV-NL63 utilizes ACE2 like SARS-CoV and SARS-CoV-2, HCoV-229E uses alanyl aminopeptidase (ANPEP). Pathogenic SARS-CoV, MERS-CoV, and SARS-CoV-2 are grouped into the lineage B or C, and of bat origin. These three viruses can cause severe diseases in humans and furthermore, COVID-19 by SARS-CoV-2 is prevalent worldwide. Its high transmission rate and incidence are notably evident among the three diseases. However, the fatality of individuals infected with MERS-CoV is significantly higher relative to that of those with SARS-CoV or SARS-CoV-2. Biological and molecular bases for the observed difference between the seasonal and pathogenic HCoVs, and also those among the pathogenic HCoVs must be determined as soon as possible.

### Replication in Cells

**FIGURE 2:**
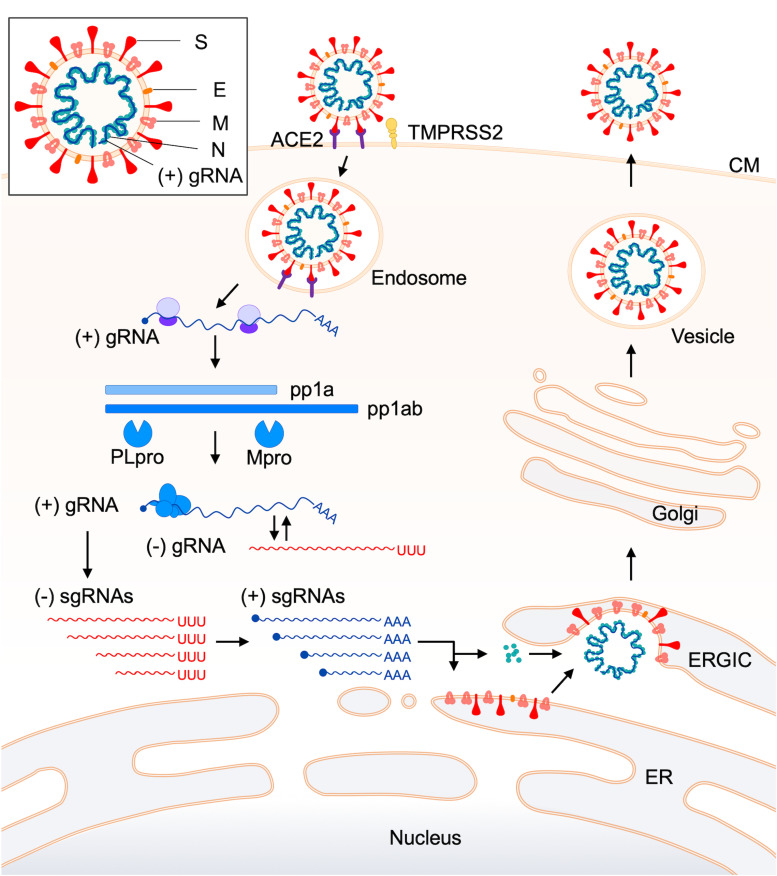
Replication cycle of coronaviruses. The replication process of coronaviruses is schematically shown from the virus attachment to target cells up to the virus release from infected cells (de Haan and Rottier, 2005; Fehr and Perlman, 2015; de Wilde et al., 2018; Oberfeld et al., 2020; Tse et al., 2020). An entry receptor and a co-factor for efficient viral entry (ACE2 and TMPRSS2, respectively, in this case) are indicated. For details of the replication steps, see the text. A schema of the coronavirus virion is also shown in a box at the top-left. Abbreviations (Fehr and Perlman, 2015; de Wilde et al., 2018): ACE2, angiotensin-converting enzyme 2; TMPRSS2, transmembrane protease, serine 2; CM, cell membrane; gRNA, genomic RNA; pp, polyprotein; PLpro, papain-like protease; Mpro, main protease; sgRNA, subgenomic RNA; ERGIC, endoplasmic reticulum-Golgi intermediate compartment; ER, endoplasmic reticulum.

CoVs utilize numerous proteins encoded by their corresponding genes (Figure 1) for replication. Accordingly, CoVs have a conserved genome structure with a high protein-coding capacity. There are 16 nsp (at most), encoded by *ORF 1a* and *ORF 1b* and generated from precursor proteins pp1a and pp1ab, for the viral RNA replication and transcription events (Figure 2). Engagement of the remarkably many proteins in the processes is probably to maintain the replication fidelity. This seems somewhat paradoxical with the highly diverse viral phenotypes observed. However, this mechanism should be essential for CoVs to survive in hostile environments. It may connote a built-in viral strategy to generate a variety of structural and accessory proteins encoded by the 3′ genomic region (Figure 1). CoVs are known to possess a unique proof-reading mechanism by the RNA-dependent RNA polymerase (RdRp) to maintain the integrity of long genomic RNA (Denison et al., 2011; Smith et al., 2013). Indeed, ongoing researches show that the mutation rate of SARS-CoV-2 is not significantly different from those of the other CoVs (Tang X. et al., 2020). The mutation rates of HCoV genomes are estimated to be moderate among those of the other single-stranded RNA virus genomes (Zhao et al., 2004; Pyrc et al., 2006; Cotton et al., 2014; Ren et al., 2015; Su et al., 2016). Therefore, it is not unreasonable to assume that the high recombination capacity of HCoVs is a major cause of the observed HCoV diversification. It deserves noting that hot spots of the high genomic recombination for SARS-CoVs are, in the higher order, *S*, *orf 8*, and *orf 3* genes (Cui et al., 2019). Remarkably, a novel accessory gene designated *orf x* has been recently identified between the *orf 6* and *orf 7* genes in the bat SARS-like CoV genomes (Ge et al., 2013; Yang et al., 2015; Hu et al., 2017; Cui et al., 2019).

The viral replication cycle in cells starts with the binding of virions to specific cellular receptors (Table 1) and ends with the release of infectious virions to the extracellular environments (Figure 2). For clarity, here, reported functions and/or activities associated with viral replication *in vitro* or *in vivo* are summarized in Table 2 for the non-structural protein (nsp) group (nsp 1–nsp 16) and in Table 3 for structural (S, E, M, and N) and accessory (ORF 3-ORF 10) proteins. CoV replication in cells is schematically outlined in Figure 2 (de Haan and Rottier, 2005; Fehr and Perlman, 2015; de Wilde et al., 2018). At the initial virus-entry step, viral S protein plays a major role in attaching to cells via the receptor. Regarding SARS-CoV, the entry step is as follows. Following virus-receptor binding, S is cleaved into two subunits S1 (receptor-binding domain, RBD) and S2 (fusion domain) by a protease such as TMPRRS2 for efficient virus entry into the cytoplasm. After endocytosis, S is further cleaved by lysosomal proteases for exposing the fusion peptide and leads to the fusion of virus envelope and endosome membrane, finally resulting in the viral RNA (+) spouting into the cytosol. Some lineage A beta-CoVs (HCoV-OC43 and HCoV-HKU1) carry another structural protein, hemagglutinin-esterase (HE), for the binding activity to sialic acids (Tables 1, 3). However, its function was being lost by the accumulation of adaptive mutations, suggesting its non-essential role in the virus entry process (Bakkers et al., 2017). The next major step is the translation and modulation of a series of non-structural proteins (Table 2), followed by the synthesis of viral genomic and various sub-genomic RNAs (van Hemert et al., 2008; Hillen et al., 2020; Romano et al., 2020; Wang Q. et al., 2020). Each sub-genomic RNA serves as mRNA for structural (S, E, M, and N) and accessory (ORF 3–ORF 10) proteins in Table 3. Notably, numerous *cis*-acting sequences/structures have been recognized in the genome (Fehr and Perlman, 2015). These contain a 5′ leader sequence, untranslated region, transcriptional regulatory sequences at the beginning of each structural and accessory gene, and 3′ untranslated region (Fehr and Perlman, 2015). Next to the viral RNA replication and RNA syntheses, viral structural and accessory proteins are produced (Figure 2). Structural proteins are inserted into the endoplasmic reticulum (ER) and move to the endoplasmic reticulum-Golgi intermediate compartment (ERGIC), where they form mature virions with viral genomic RNA. Subsequently, progeny virions in vesicles are transported to the cell surface and released to the outside environments. Although a general picture of the CoV replication steps is acquired, biological and molecular biological studies in more detail are still required, especially for the accessory proteins (Table 3) and for the functional interactions of various viral proteins. Generally, while the pathogenic CoVs (SARS-CoV, MERS-CoV, and SARS-CoV-2 in Table 1) grow well in cultured cell lines, the seasonal CoVs (HCoV-NL63, HCoV-229E, HCoV-OC43, and HCoV-HKU1 in Table 1) propagate very poorly or negligibly. Also, systemic and comparative studies on the cellular tropism of various HCoVs have not yet been performed. As such, numerous projects at the cellular level remain to be carried out to understand biological and molecular bases for the HCoV virology.

**TABLE 2:** Coronaviral non-structural proteins encoded by the conserved genomic 5′ region.

Proteins	Function/activity and comments
nsp 1	Suppresses the host innate immune response by degrading host mRNA degradation, blocking host translation, antagonizing IFN, and blocking STAT1 phosphorylation.
nsp 2	Is dispensable for viral replication in cultured cells. Interacts with cell proteins prohibitin 1 (PHB1) and PHB2. May disrupt the host signaling process.
nsp 3	Encodes one or two papain-like proteases (PLpro) that cleave the nsp 1/2, nsp 2/3, and nsp 3/4 boundaries in pp1a and pp1ab proteins. Large, multi-domain/multi-activity (the interaction with N protein, promotion of cytokine expression, blockade of host innate immunity, etc.) transmembrane protein.
nsp 4	Is a transmembrane protein. May be a scaffold protein for virus-induced intracellular structure, double-membrane vesicles (DMVs), but is dispensable for viral replication in cultured cells.
nsp 5	Is a main serine type protease (Mpro) that processes the 11 cleavage sites in pp1a and pp1ab proteins other than those by PLpro. Also called 3C-like protease (3CLpro).
nsp 6	Is a transmembrane protein. Function unknown. May be a scaffold protein for DMVs like nsp 4 protein.
nsp 7	Is a cofactor for an RNA-dependent RNA polymerase (RdRp) protein nsp 12. Forms a complex with nsp 8 and RdRp proteins to act as a processivity clamp for RNA polymerase. Antagonizes IFN by an undescribed molecular mechanism.
nsp 8	Is a cofactor for nsp 12 RdRp protein. Forms a complex with nsp 7 and RdRp proteins to act as a processivity clamp for RNA polymerase.
nsp 9	Function unknown. Binds to RNA and may interact with nsp 8 protein. Considered to be important for the replicase-transcriptase complex (RTC).
nsp 10	Is the cofactor for nsp 14 and nsp 16 proteins. Forms heterodimer with these proteins and thereby stimulates both viral 3′–5′ exoribonuclease (ExoN) and 2′-O-ribose methyltransferase (2-O-MT) activities.
nsp 12	Is an RdRp and forms a complex with nsp 7 and nsp 8 proteins.
nsp 13	Has a variety of enzymatic functions including NTPase, dNTPase, RNA 5′-triphosphatase, RNA helicase, and DNA helicase activities.
nsp 14	Has guanine-N7 methyltransferase (N7 MTase) and ExoN activities. While N7 MTase adds 5′ cap to viral RNAs, ExonN plays a critical role in proofreading viral genomes.
nsp 15	Is uridylate-specific viral endoribonuclease (NendoU). Antagonizes IFN by an undescribed molecular mechanism.
nsp 16	Has 2-O-MT activity. Shields viral RNAs from the melanoma differentiation association protein 5 (MDA5, an intracellular virus sensor) recognition by modifying the cap of viral RNAs.

*For details, see the relevant review articles (Totura and Baric, 2012; Fehr and Perlman, 2015; de Wilde et al., 2018; Hillen et al., 2020; Oberfeld et al., 2020; Park and Iwasaki, 2020; Romano et al., 2020; Tang D. et al., 2020; Tse et al., 2020; Wu A. et al., 2020). All CoV genomes determined so far contain the 16 ORFs in their 5′ genomic regions. It is uncertain for now as to whether a very small protein of nsp 11 really exists by itself and plays a functional role in viral replication (Chan et al., 2020). nsp, non-structural protein; IFN, interferon; NTPase, nucleoside triphosphatase; dNTPase, deoxynucleoside triphosphatase.*

**TABLE 3:** Coronaviral structural and accessory proteins encoded by the variable genomic 3′ region.

Proteins	Function/activity and comments
S: spike (structural protein)	Is a type 1 fusion glycoprotein present on the virion surface as a homotrimer. Mediates virus attachment to the host cellular receptor and subsequent virus entry into host cells. Is triggered for membrane-fusion activity upon cleavage into S1 and S2 subunits by the cell protease.
E: envelope (structural protein)	Is a transmembrane protein and present in a small quantity within the virion. Is highly divergent but its structure is conserved. Facilitates the virus assembly and release from cells. Has the ion channel activity and affects viral pathogenicity.
M: membrane (structural protein)	Is the most abundant virion structural protein with three transmembrane domains. Directs most protein-protein interactions (with E, N, S) required for the assembly of coronaviruses. Antagonizes various processes of the antiviral host immune response.
N: nucleocapsid (structural protein)	Is the only viral protein in the nucleocapsid and binds to RNA including the genomic packaging signal. Also binds to nsp 3 and M proteins to promote the formation of infectious virions. Counteracts various steps of antiviral host immune response.
ORF 3 to ORF 10* (accessory proteins)	ORF 3b/ORF 6 (SARS-CoV-2) and ORF 3b (bat SARS-like coronavirus) suppress the host innate immune response by antagonizing IFN in different ways. ORF 4a/4b/5 (MERS-CoV) suppress the host innate immune response by blocking IFN signaling through distinct routes. ORF 7 (TGEV) counteracts the host’s antiviral response by modulating host cell translation. MERS-CoV, more sensitive to IFN than SARS-CoV, lacks ORF 6 and ORF 7 homologs. ORF 8b/8ab (SARS-CoV) inhibit IFN response in host cells. Overall, coronaviral accessory proteins appear to be dispensable for viral replication in cultured cells, but most likely to play a solid and critical role in counteracting the host innate anti-viral immunity through distinct signaling routes.

Some lineage A beta-CoVs (HCoV-OC43 and HCoV-HKU-1 in Table 1) and related bovine CoVs also have a hemagglutinin-esterase (HE) protein on the virion surface (Forni et al., 2017). For reference, see the relevant review articles (de Haan and Rottier, 2005; Totura and Baric, 2012; Fehr and Perlman, 2015; Forni et al., 2017; de Wilde et al., 2018; Oberfeld et al., 2020; Park and Iwasaki, 2020; Tang D. et al., 2020; Tse et al., 2020; Wu A. et al., 2020). ORF, open reading frame; IFN, interferon; TGEV, transmissible gastroenteritis virus (porcine virus). *Because there are extensive accessory ORF protein divergences and also because relevant scientific reports are limited, statements on the function/activity are individually described for each protein in the right column.

### Host Responses and Viral Adaptations

While scientifically confirmed knowledge on viral replication and related issues *in vitro* underpins the understanding of the complicated nature of pathogenic CoVs, knowing various responses of hosts to the virus infection may be critically important as well to solve the present scientific issues in the laboratories. Because the virus infection process sharply reflects the halfway and final results of viral conflict or interaction with hosts, extensive studies at the levels of the cell, individual, and population are essential. In this regard, a number of articles regarding SARS/MERS (Totura and Baric, 2012; Channappanavar et al., 2016; de Wit et al., 2016; Su et al., 2016; Park and Iwasaki, 2020) and also regarding COVID-19 (Biswas et al., 2020; Bost et al., 2020; Broggi et al., 2020; Brouwer et al., 2020; Cao et al., 2020; Giamarellos-Bourboulis et al., 2020; Gordon et al., 2020; Grifoni et al., 2020; Hansen et al., 2020; Ju et al., 2020; Kadkhoda, 2020; Kim D. et al., 2020; McKechnie and Blish, 2020; Oberfeld et al., 2020; Ong et al., 2020; Polycarpou et al., 2020; Robbiani et al., 2020; Shi R. et al., 2020; Sun J. et al., 2020; Tang D. et al., 2020; Tay et al., 2020; Vabret et al., 2020; Wilk et al., 2020; Xu et al., 2020; Ye et al., 2020; Zhang et al., 2020; Zhou P. et al., 2020; Zhou Z. et al., 2020; Ziegler et al., 2020; Zohar and Alter, 2020) have been published and have provided detailed information on the epidemiology of the diseases, pathophysiological nature of the disease, clinical features of the patients, viral and host factors associated with the infection, the clinical symptoms, host innate immune responses, neutralizing antibodies against viruses, and so on. Scientific dealing with these huge amounts of information is surely a next, important, and challenging step for experimental virologists currently working on SARS-CoV-2.

With the unique genome organization and the genomic sequence characteristics described above, CoVs have a highly flexible potential to mutate in fluxing environments. Generally, adaptive mutations can occur in an amino acid-dependent and/or in a nucleotide-dependent manner to a high degree for RNA viruses. Moreover, drastic alterations of the genome organization such as the gain and loss of genes, which are frequently observed for CoVs (Forni et al., 2017), would give the concerned virus the potential to fiercely change its biological properties. Given the diverse receptor usage (Table 1; Fehr and Perlman, 2015; Forni et al., 2017; de Wilde et al., 2018; Tang D. et al., 2020), high replicative ability (Yount et al., 2000, 2002, 2003; Scobey et al., 2013; Thao et al., 2020; Xie et al., 2020), broad host tropism (Su et al., 2016; Forni et al., 2017; Cui et al., 2019; Tang D. et al., 2020), ongoing inter- and intra-species transmission among animals (Lau et al., 2005; Li et al., 2005a; Woo et al., 2012; Huang et al., 2013; Brook and Dobson, 2015; Zhou et al., 2018; Han et al., 2019; Wang and Anderson, 2019; Letko et al., 2020b), and occasional zoonotic transmission to humans (Table 1; Drosten et al., 2003; Fouchier et al., 2003; Ksiazek et al., 2003; Zhong et al., 2003; Zaki et al., 2012; Lu et al., 2020; Wu A. et al., 2020; Zhu et al., 2020), CoVs are tractable and outstanding targets for experimental studies on the recombination, mutation, adaptation, and evolution. One clue that may be attributed to the adaptations and diversifications of HCoVs is the possible switching of receptor usages. As shown in Table 1, there are a variety of receptors for HCoVs: ACE2, ANPEP/CD13, DPP4/CD26, and maybe some more. Therefore, these types of receptor switchings occur frequently during the evolution of CoVs. Interestingly, S protein, the spike protein on the virion surface that primarily and directly interacts with host cell receptors, contains a recombination hotspot (Hon et al., 2008; Wu et al., 2016; Hu et al., 2017; Cui et al., 2019), which has been positively selected (Lau et al., 2015; Forni et al., 2017). Thus, the high frequency of RNA recombination observed in CoVs (Lai et al., 1985; Keck et al., 1987; Lai, 1992; Nagy and Simon, 1997), possibly through the secondary structure and replication stalling of RNA and the non-processive replicase-driven template switching mechanism (Lai, 1992; Rowe et al., 1997), supports that the recombination at this hotspot is important for adaptations to new host types, and the diversification/evolution of CoVs. Indeed, there is evidence for *in vivo* recombination between animal and human CoVs (Wu et al., 2003). Also, there are a number of reports that demonstrate the adaptive recombination/mutations in the *S* gene of HCoV-229E, SARS-CoV, MERS-CoV, and SARS-CoV-2 (Li et al., 2005b; Wong et al., 2017; Letko et al., 2018, 2020a; Gussow et al., 2020), as described above for the *HE* gene of lineage A beta-CoVs (HCoV-OC43 and HCoV-HKU1). Of note, a growth-enhancing mutation has been found in the S protein of SARS-CoV-2 during this pandemic (Korber et al., 2020). CoV S protein is a key viral factor that primarily shoulders the host cell and species tropism, one of the critical viral properties, and thus, its biologically significant variations would influence much the viral phenotype. Of note here, the structure-based phylogenetic analysis of the receptor-binding S1 domain (Forni et al., 2017; Ng et al., 2020) and also the extensive systemic study on the receptor-usage and infection ability to cell types of different species (Letko et al., 2020a) have suggested the presence of an unknown receptor(s) for HCoVs. The current seven species of HCoVs are most likely to have appeared through evolution via multiple complicated receptor switching events. Totally and historically, CoV adaptive determinants for tropism, replication ability and/or pathogenicity have not yet been systemically analyzed except for S protein. In particular, the possible involvement of the HCoV accessory proteins in these fundamental virus properties is poorly studied in a demonstrative manner so far. Extensive studies in this direction need to be urgently carried out.

## Reverse Genetics Technology for Animal and Human Coronaviruses

**FIGURE 3:**
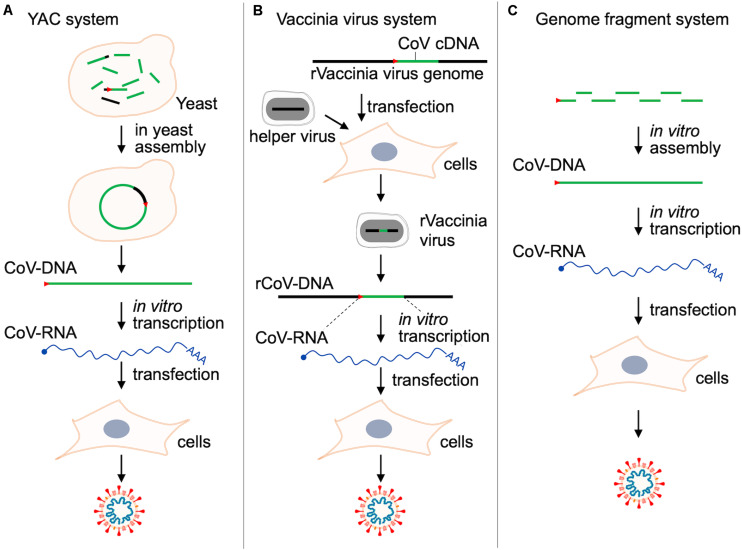
Reverse genetics systems for studies on CoVs. Outlines of the three major methods to produce CoVs by RNA transfection are shown. For details, see Thao et al. (2020) for panel **(A)**, Thiel et al. (2001) for panel **(B)**, and Hou et al. (2020); Xie et al. (2020) for panel **(C)**. The BAC system (Table 4) is essentially quite similar with that in panel **(A)**, but a complete full-length CoV-DNA must be constructed *in vitro* as BAC before transformation into bacteria and the following preparation of plasmid DNA (Almazán et al., 2000). Red arrow heads in this figure indicate the T7 promoter sequence. r, recombinant.

The most frequently and widely utilized experimental system for analytical studies on human/animal viruses would be the reverse genetics. To study biology and molecular biology of viruses, reverse genetic systems are almost prerequisite methods in the current virology. With the aid of the genetic system, we can readily perform a series of mutational functional studies on any coding or non-coding regions of any genes, expecting to have reproducible experimental data on a solid basis. We can apply it, other than the orthodox functional studies, to a wide variety of research projects such as those on the effects of spontaneously occurring natural variations, adaptive mutations in relation to virus evolution, interactions of multiple viruses, prediction of viral drug/vaccine resistance, and so on. The reverse genetics is the most powerful and superior method in today’s virology. However, mostly due to their extraordinarily large RNA genomes, it was quite difficult to establish valid reverse genetics systems for CoVs. It is hard to stably maintain such a long genome in the DNA vectors in the microbes for genetics, and sometimes the cloned DNAs contain some toxic sequences to the microbes concerned. In addition, in most cases, cloned DNAs need to be transcribed *in vitro* into RNAs for RNA (+) viruses like CoVs before experimental use (for RNA transfection). Researchers thus have come up with various resources to make the methodology easy to use (Almazán et al., 2014). As listed in Table 4, the reverse genetics methods for various CoVs are technically divided into four types: BAC (bacterial artificial chromosome), YAC (yeast artificial chromosome), a large DNA virus vaccinia, and plasmid assembly systems. Outlines of the methods to produce genetically engineered CoVs by RNA transfection are schematically presented in Figure 3.

**TABLE 4:** Reverse genetics systems for studies on various mammalian coronaviruses.

Viruses (hosts)	Methods for reverse genetics	References
TGEV (swine)	Bacterial system. BAC (low-copy number plasmid) cDNA clone encoding an infectious viral RNA genome.	Almazán et al., 2000
TGEV (swine)	Bacterial system. Full-length cDNA clone by assembling a series of subclones.	Yount et al., 2000
HCoV-229E (human)	Vaccinia virus system. Full-length cDNA clone in the vaccinia viral genome.	Thiel et al., 2001
MHV (mouse)	Bacterial system. Full-length cDNA clone by assembling a series of subclones.	Yount et al., 2002
SARS-CoV (human)	Bacterial system. Full-length cDNA clone by assembling a series of subclones.	Yount et al., 2003
MERS-CoV (human)	Bacterial system. Full-length cDNA clone by assembling a series of subclones.	Scobey et al., 2013
Chimera (mouse)	Bacterial system. Full-length cDNA clone by assembling a series of subclones. Chimera of mouse-adapted SARS-CoV and bat-CoV.	Menachery et al., 2015
WIV1-CoV (bat)	Bacterial system. Full-length cDNA clone by assembling a series of subclones. SARS-like WIV1-CoV.	Menachery et al., 2016
MERS-CoV (human)	Bacterial system. BAC clone manipulated by the bacteriophage λ Red recombination system.	Muth et al., 2017
SARS-CoV-2 (human)	Bacterial system. Full-length cDNA clone by assembling a series of subclones.	Xie et al., 2020
SARS-CoV-2 (human) MERS-CoV (human) MHV (mouse)	Yeast system. YAC cDNA clone encoding an infectious viral RNA genome.	Thao et al., 2020
SARS-CoV-2 (human)	Bacterial system. Full-length cDNA clone by assembling a series of subclones.	Hou et al., 2020

*TGEV, transmissible gastroenteritis virus; BAC, bacterial artificial chromosome; MHV, mouse hepatitis virus; YAC, yeast artificial chromosome.*

We have summarized these systems chronologically in Table 4. In a pioneer study, a low-copy number BAC cDNA clone encoding a full-length viral RNA genome was used to generate the infectious transmissible gastroenteritis virus (TGEV) (Almazán et al., 2000). This paper successfully demonstrated that viral tropism and virulence can be modified *in vitro*. Then, Baric and his team generated an infectious TGEV construct (Yount et al., 2000). They initiated and developed a novel technology by which a complete clone was made through assembling several subclones in order. They successively and successfully generated full-length infectious clones of mouse hepatitis virus (MHV) (Yount et al., 2002), SARS-CoV (Yount et al., 2003), MERS-CoV (Scobey et al., 2013), mouse-adapted SARS-CoV (Menachery et al., 2015), bat-derived WIV1-CoV (Menachery et al., 2016), and finally SARS-CoV-2 (Hou et al., 2020). Furthermore, the methods other than those described above were proposed for genetic studies on CoVs. These are a vaccinia-based and a new BAC system for HCoV-229E (Thiel et al., 2001) and MERS-CoV (Muth et al., 2017), respectively. In 2020, complying with researchers’ expectations and strong requests, two reverse genetics methodologies for SARS-CoV-2, i.e., the assembly (Hou et al., 2020; Xie et al., 2020) and the YAC (Thao et al., 2020) systems, have been reported. The authors of the three articles (Table 4) have demonstrated that their systems are useful for various functional analyses on SARS-CoV-2. Of note, both systems can be or were applied to the other CoVs such as MHV, SARS-CoV, MERS-CoV, HCoV-229E, and HCoV-HKU1. Also, full-length constructs carrying a marker reporter gene have been constructed for MHV, MERS-CoV, SARS-CoV, and SARS-CoV-2 (Hou et al., 2020; Xie et al., 2020; Thao et al., 2020). Importantly and notably, it has been successfully demonstrated that the specific infectivity of SARS-CoV-2 for the respiratory tract region is determined in an ACE2-dependent manner (Hou et al., 2020). Furthermore, it has been shown that ciliated airway cells and AT-2 cells (type II pneumocytes, constituents of adult alveolar epithelium) are the primary targets for SARS-CoV-2 (Hou et al., 2020). Researchers can follow one of these or all of them as a powerful analyzing tool for SARS-CoV-2 biology and molecular biology, depending on their scientific preference and/or experience. By using the experimental systems described above (Figure 3), researchers will be able to perform a systemic analysis on SARS-CoV-2 and related issues in a demonstrative manner. Besides studies on highly pathogenic HCoVs, comparative functional analyses by the reverse genetics system using seasonal HCoVs and animal CoVs closely related to HCoVs may be important to systematically understand the biology of HCoVs. The comparative molecular virology is a legitimate approach of experimental virology today. Coupled with *in vitro* laboratory and *in vivo* animal experiments, the reverse genetics system (Figure 3) would prove a real worth (Totura and Baric, 2012; Almazán et al., 2014; Bakkers et al., 2017; Wong et al., 2017; Letko et al., 2018, 2020a; Bao et al., 2020; Chandrashekar et al., 2020; de Wit et al., 2020; Hou et al., 2020; Jiang et al., 2020; Lakdawala and Menachery, 2020; Shi J. et al., 2020; Sun S.-H. et al., 2020; Thao et al., 2020; Williamson et al., 2020; Xie et al., 2020; Yu et al., 2020).

## Conclusion: Future Studies in a Demonstrative and Perspective Manner

In the present context that so many review articles on CoVs are being published, we emphasize our review as containing new concepts and viewpoints regarding the corona-virology based on the CoV unique and critical properties so far reported. We do care about the diversification of coronaviruses and care for the underlying molecular mechanisms by which the highly diverse phenotypes of CoVs are created. Needless to mention, the principal cause lies in the CoV genome itself. Its unusually long RNA genome and complicatedly regulated expression system certainly constitute a foundation for ever-changing appearance of CoVs. In addition, plenty of circumstantial evidence fully indicates that the environmental factors strongly assist its diversifying potential. Thus, we need to eagerly engage or be interested in both of the laboratory/clinical research and the fieldwork (Adachi et al., 2020).

On the basis of the fundamental studies consistently continued by the CoV investigators and the stimulating and thought-provoking experience in the recent three CoV outbreaks, researchers must prepare for the future in the medium or long term. We long have been involved in molecular genetic studies of human and simian immunodeficiency viruses (HIV/SIVs) as described above. Despite considerable differences in their biological and molecular biological properties, research concepts, strategies, and tools are common between the two virus species, CoV and HIV/SIV (Adachi, 2020). More than anything, we have to handle the global disease-causing nature of the two viruses, SARS-CoV-2 and HIV-1. We, as experimental virologists, analyze the CoVs in a solidly demonstrative and perspective manner by utilizing the reverse genetics methodology and related technology (Table 4 and Figure 3) as exactly is the case for studies on HIV/SIVs, and use the various animal model systems available (Bao et al., 2020; Chandrashekar et al., 2020; de Wit et al., 2020; Jiang et al., 2020; Lakdawala and Menachery, 2020; Shi J. et al., 2020; Sun S.-H. et al., 2020; Williamson et al., 2020; Yu et al., 2020) when necessary as a part of experimental virology. Having a bird’s-eye view is essential for studies on viruses of this kind. Finally, because numerous papers on COVID-19/SARS-CoV-2 have been published on a day-by-day basis in 2020, we sort out the latest publications (original, review, and other types of articles so far published in 2020, as of June), and cite them here in parentheses according to the four categories below. These articles are generally important for CoV research and worth referencing from our standpoint. Although drugs, neutralizing antibodies, and vaccines against SARS-CoV-2 and related viruses are urgently important, of course, in this challenging time, we do not take up the issue in this review. Refer to the articles below, instead.

### Drugs, Neutralizing Antibodies and Vaccines

(Baum et al., 2020; Brouwer et al., 2020; Burton and Walker, 2020; Dai et al., 2020; de Wit et al., 2020; Diamond and Pierson, 2020; Gordon et al., 2020; Hansen et al., 2020; Hassan et al., 2020; Jin et al., 2020a, b; Kim E. et al., 2020; Rogers et al., 2020; Tang D. et al., 2020; Tse et al., 2020; Wang N. et al., 2020; Wec et al., 2020; Williamson et al., 2020; Wu Y. et al., 2020; Yu et al., 2020; Zhou and Zhao, 2020).

### Host Responses to Infection and Clinical Outcomes

(Biswas et al., 2020; Bost et al., 2020; Broggi et al., 2020; Brouwer et al., 2020; Cao et al., 2020; Davies et al., 2020; Giamarellos-Bourboulis et al., 2020; Gordon et al., 2020; Grifoni et al., 2020; Hansen et al., 2020; Ju et al., 2020; Kadkhoda, 2020; Kim D. et al., 2020; Long et al., 2020; McKechnie and Blish, 2020; Oberfeld et al., 2020; Ong et al., 2020; Park and Iwasaki, 2020; Polycarpou et al., 2020; Robbiani et al., 2020; Shi R. et al., 2020; Subbarao and Mahanty, 2020; Tang D. et al., 2020; Tay et al., 2020; Vabret et al., 2020; Wilk et al., 2020; Xu et al., 2020; Ye et al., 2020; Zhang et al., 2020; Zhou P. et al., 2020; Zhou Z. et al., 2020; Ziegler et al., 2020; Zohar and Alter, 2020).

### Viral Basic Properties, Adaptations, and Diversifications

(Andersen et al., 2020; Baum et al., 2020; Biswas et al., 2020; Chan et al., 2020; Gussow et al., 2020; Hillen et al., 2020; Hoffmann et al., 2020; Hou et al., 2020; Jaimes et al., 2020; Kim D. et al., 2020; Letko et al., 2020a, b; Li et al., 2020; Matsuyama et al., 2020; Ng et al., 2020; Oberfeld et al., 2020; Prather et al., 2020; Qi et al., 2020; Rogers et al., 2020; Romano et al., 2020; Shang et al., 2020; Su et al., 2016; Sun J. et al., 2020; Tang D. et al., 2020; Thao et al., 2020; Walls et al., 2020; Wang Q. et al., 2020; Wec et al., 2020; Wrapp et al., 2020; Wu A. et al., 2020; Xie et al., 2020; Ye et al., 2020; Ziegler et al., 2020).

### Host Animals and Animal Experiments

(Bao et al., 2020; Chandrashekar et al., 2020; de Wit et al., 2020; Hansen et al., 2020; Hassan et al., 2020; Jiang et al., 2020; Lakdawala and Menachery, 2020; Letko et al., 2020b; Rogers et al., 2020; Shi J. et al., 2020; Sun S.-H. et al., 2020; Williamson et al., 2020; Yu et al., 2020; Zhou and Zhao, 2020).

## Author Contributions

TK, AA, and MN conceived the idea. TK depicted the figures. AA and SA made a draft. TK, ND, and MN reviewed it and discussed its content. AA and MN wrote a final manuscript. All authors approved its submission.

## Conflict of Interest

The authors declare that the research was conducted in the absence of any commercial or financial relationships that could be construed as a potential conflict of interest.
